# Bioinspired Superhydrophobic Surfaces via Laser-Structuring

**DOI:** 10.3389/fchem.2020.00835

**Published:** 2020-10-16

**Authors:** Monan Liu, Mu-Tian Li, Shuai Xu, Han Yang, Hong-Bo Sun

**Affiliations:** ^1^Department of Condensed Matter Physics, College of Physics, Jilin University, Changchun, China; ^2^State Key Laboratory on Integrated Optoelectronics, College of Electronic Science and Engineering, Jilin University, Changchun, China

**Keywords:** bioinspired surfaces, superhydrophobic surfaces, laser structuring, femtosecond lasers, graphene

## Abstract

Bioinspired superhydrophobic surfaces are an artificial functional surface that mainly extracts morphological designs from natural organisms. In both laboratory research and industry, there is a need to develop ways of giving large-area surfaces water repellence. Currently, surface modification methods are subject to many challenging requirements such as a need for chemical-free treatment or high surface roughness. Laser micro-nanofabrications are a potential way of addressing these challenges, as they involve non-contact processing and outstanding patterning ability. This review briefly discusses multiple laser patterning methods, which could be used for surface structuring toward creating superhydrophobic surfaces.

## Introduction

Bioinspired surfaces are surfaces with artificial micro-nanostructures that mimic functional biological structures in natural organisms. They exploit the diverse functions of the natural counterparts, who have acquired rich biological features and advantages during their long-term evolution and adaptation to nature. Among these biological features, surface wettability is a key property due to its extensive possible applications. There are a number of natural surfaces that show properties such as superhydrophobicity as well as the ability to self-clean, including rose petals, reed leaves, and even the skin of some animals (Martinez-Calderon et al., 2016a). It has been fully demonstrated that such superhydrophobicity is decided by both surface chemistry and unique surface micro-nanostructures (Florian et al., 2018), which provides the basic strategy for developing bioinspired superhydrophobic surfaces. In rose petals, the surfaces consist of densely packed microhills covered by a large number of microtrenches, whose wetting regime corresponds to the Wenzel state (Feng et al., 2011). In another example, lotus leaves, both papilla-like microstructures and ceraceous-covered nanoscale textures form hierarchical micro-nanostructures and result in water-repellent behavior, whose wetting regime corresponds to the Cassie-Baxter state (Fan et al., 2015). During recent decades, manipulation of surface wettability, especially obtaining superhydrophobic surfaces, has proved to have potential applications in a series of fields, including for self-cleaning materials (Vorobyev and Guo, 2015), corrosion improvement (Su and Yao, 2014), and oil-water separation (Liu et al., 2017). Recently, it has also been found that such superhydrophobic surfaces may shed light on cutting-edge fields such as developing anti-bacterial properties (Bremus-Koebberling et al., 2012; Schieber et al., 2017) and de-icing aircraft (Jung et al., 2011; Heydari et al., 2013).

Based on this research, both chemical and geometrical modifications have been adopted and used to obtain superhydrophobic surfaces. This can involve coating target surfaces with a hydrophobic layer (Kam et al., 2012; Weisensee et al., 2014), or treating them by wet etching (Weibel et al., 2010). The main problem with chemical surface treatment is the unavoidable chemical residue that sometimes remains on target surfaces, which may be toxic or even negatively affect the device performance under certain temperature and pressure conditions. In pursuit of extreme surface roughness, a variety of micro-nanofabrication techniques can be adopted, including molding (Zhao et al., 2008), electrodeposition (Liu et al., 2016), and photolithography (Limongi et al., 2015). Among these techniques, laser structuring is known for its non-contacting, wide material-adaptability, and advanced 3D patterning ability. The first work to demonstrate laser-structured superhydrophobic surfaces dates back to 2006 when silicon surface was textured with microspikes by 800-nm femtosecond (fs) lasers under the flow of a reactive gas SF_6_ (Baldacchini et al., 2006). Together with a 3-h chemical modification by (heptadecafluoro-1,1,2,2-tetrahydrodecyl) trichlorosilane [CF_3_(CF_2_)_7_CH_2_CH_2_SiCl_3_], the two-step treatment turned the silicon surface to superhydrophobic. With this increased laser fluence, hierarchical structures were also obtained on a silicon substrate by similar one-step processing via fs lasers under a reactive atmosphere (Zorba et al., 2008). The resulting microconicals and nanoprotrusions appeared all over the microstructure surfaces and successfully mimicked the surface structures of a lotus–leaf, realizing similar water–repellent properties. The formation of the microstructures was ascribed to the cooperation of capillary waves and laser-induced etching (Vorobyev and Guo, 2013). With more explorations in this scheme, it has been found that fs laser direct-writing can induce four kinds of self-organized structures: ripples, microgrooves, microspikes, and hierarchical complex structures (Stratakis et al., 2020). These micro-nanostructures give rise to surface textures as well as hydrophobicity/superhydrophobicity, and morphologies are tunable by laser parameters such as laser fluence, pulse duration, and repetition rate (Vorobyev and Guo, 2013; Stratakis et al., 2020). A series of bioinspired surfaces that highly mimic functional natural surfaces have been realized by flexibly utilizing multiple laser-processing methods (Stratakis et al., 2020). Surfaces have been created by fs laser direct-writing, which have also proved to have applications in a variety of fields, including self-cleaning, stimuli-responsive surfaces (light, electro, pH, etc.) (Papadopoulou et al., 2009b; Stratakis et al., 2010, 2011), cell adhesion (Ranella et al., 2010), tissue engineering (Papadopoulou et al., 2009a), etc. (Vorobyev and Guo, 2013; Stratakis et al., 2020). Recently, it has also been demonstrated that tuning fs-laser polarization could induce hierarchical surface structures on Ni, due to the unique donut-like profile of radically- and azimuthally- polarized fs lasers (Skoulas et al., 2017). In order to avoid chemical pollution to target surfaces, one can focus on direct laser-structuring methods, which are laser-induced periodic surface structures (LIPSS), direct laser-interference patterning (DLIP), and direct laser writing (DLW).

Herein, we have briefly reviewed recent achievements in obtaining superhydrophobic surfaces via the laser-structuring methods. According to the diverse surface properties of the target materials as well as the varied requirements of different applications, a single technique or even multi-techniques can be used to define functional surface micro-nanostructures for manipulating surface wettability. This covered the technological features of the three distinctive laser-structuring methods and the resulting surface structure morphologies due to laser-enabled geometrical modifications. Especially, the flexible method combinations involved in obtaining complex hierarchical surface structures for extreme surface roughness as well as water-repellent property.

## Laser Induced Periodical Surface Structures (LIPSS)

**Figure 1:**
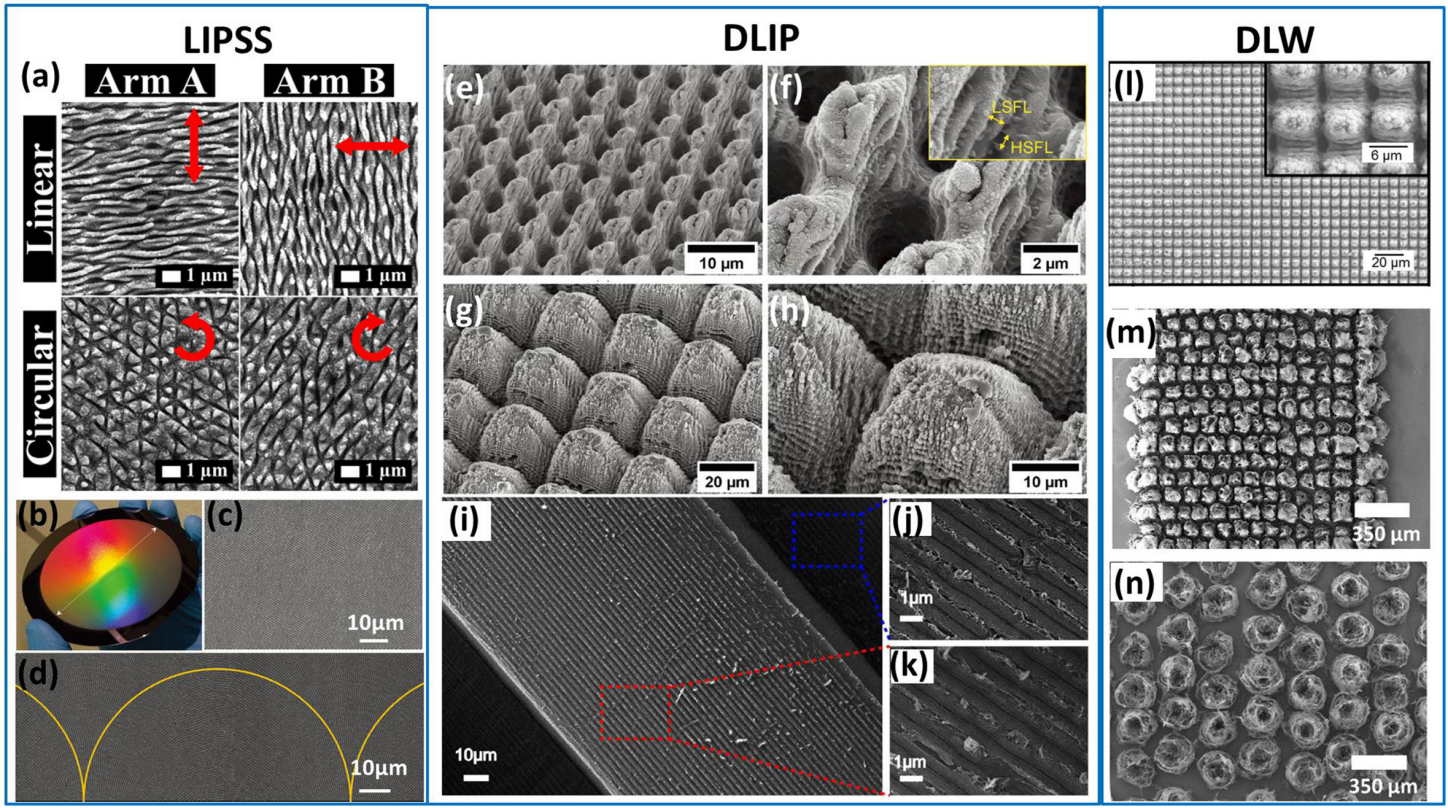
Bioinspiredsuperhydrophobic surfaces via laser-structuring. **(a)** LIPSS formed on stainless steel by femtosecond lasers with either linear or circular polarization; **(b–d)** Large-area LIPSS formed on silicon; Superhydrophobic surfaces structured by DLIP on **(e–h)** Ti64 (together with LIPSS) and **(i–k)** on reduced graphene oxide; Superhydrophobic surfaces structured by DLW on **(l)** Al2024 (combined with DLIP) and **(m,n)** on polyimide films. Reproduced from Fraggelakis et al. (2019) with permission of Elsevier. Reproduced from Wang et al. (2017) with permission of Nature. Reproduced from Vercillo et al. (2020) with permission of WILEY-VCH. Reproduced from Jiang et al. (2018) with permission of the American Chemical Society. Reproduced from Milles et al. (2019) with permission of Nature. Reproduced from Nasser et al. (2020) with permission of Elsevier.

Laser induced periodical structures (LIPSS) are self-organized structures commonly found when laser irradiation on material surfaces is concerned. Previous studies have found them on a variety of materials, covering metal, semiconductors, and dielectrics (Wang et al., 2017; Ahsan et al., 2020; Gao et al., 2020; Yang et al., 2020). LIPSS are usually ripple-like periodical structures with a spatial period Λ close to or smaller than the wavelength λ of incident lasers. They can be further classified into low spatial frequency LIPSS (LSFL, Λ ~ λ) and high spatial frequency LIPSS (HSFL, Λ ~ λ/3) (Wang et al., 2017). The structure orientation is decided by laser polarization, either parallel or perpendicular (most cases for LSFL) to the polarization direction (Wang et al., 2017). Surfaces modified with LIPSS have shown potential anti-bacterial functions and could also be used in cell proliferation (Rebollar et al., 2008; Martinez- Calderon et al., 2016b; Giannuzzi et al., 2019). Moreover, the wavelength- or subwavelength-scale of LIPSS makes them ideal for acquiring hierarchical micro-nanostructures and reinforcing superhydrophobicity. So far, the formation mechanisms of LIPSS are still under debate, with proposed models such as interference between incident and scattered light waves or excited surface Plasmon polaritons (Buividas et al., 2014; Li et al., 2019) and local-defect-induced plamas (Taylor et al., 2008). Despite these debates, the structure orientation, spatial period, and structural dimensions of LIPSS can be tuned by controlling key parameters of the processing system, including incident wavelength, polarization, number of exposures, and so forth (Fraggelakis et al., 2019). This fine-tuning can give rise to patterning flexibility and surface functionalities not limited to superhydrophobicity (Jiang et al., 2014). Multi-pulse irradiations by fs lasers with circular polarization and tunable interpulse delay were performed on stainless steel and tungsten to generate two-dimensional (2D) LSFL (Liu et al., 2018; Romano et al., 2018; Fraggelakis et al., 2019), as shown by Figure 1a. Both HSFL and LSFL could be generated simultaneously by such bursts of 1,030-nm fs lasers with picosecond (ps) delays when the burst number was above four (Giannuzzi et al., 2019). LIPSS modification has also been performed on other metal materials for obtaining superhydrophobic surfaces, such as copper (Liu et al., 2017). Triply hierarchical structures (micro-trenches, nanoripples, and nanoparticles) were obtained on copper foil surface by the irradiation of 800-nm fs lasers with a repetition rate of 1 kHz (Allahyari et al., 2019). The structure complexity effectively reinforced the hydrophobicity of the copper foil surface, increasing the water contact angle (WCA) from 100° to 160°. Moreover, large-area LIPSS were also realized by a transversely-elongated focal spot with the help of a cylindrical lens. The scanning speed was raised to 50 mm/s and wafer-scale LSFL could be achieved on the silicon substrate (Wang et al., 2017), as shown by Figures 1b–d.

## Direct Laser Interference Patterning (DLIP)

DLIP is making two or more coherent laser beams interfere such that periodical micro-nanostructures can be directly defined on substrates due to interference pattern. The micro-nanostructures that are formed correspond to the local energy (or amplitude) distribution. Diverse micro-nanostructures can be obtained by tuning the number and included angles of the laser beams, the laser wavelength, and polarization, and the number of exposures, etc. (Abid et al., 2017). The wavelength of the obtained periodical structures is decided by both the incident laser wavelength λ and included angle θ between the two laser beams,

(1) $$\begin{array}{r} \Lambda \text{=}\frac{\lambda }{2\sin\left(\theta /2\right)} \end{array}$$

Usually, for a specific laser source with picosecond or femtosecond pulses, it is convenient to tune the angle to θ for obtaining patterns with different wavelengths. DLIP is of the highest throughput among the three laser-structuring techniques [0.9 m^2^ min^−1^ (Lang et al., 2016)], since it can process a sub-milliscale region with one single shot. One-dimensional micrograting structures generally need one exposure and two-dimensional micrograting structures (i.e., micropillar arrays) need two exposures with a holding stage rotated by a certain angle (such as 60°or 90°) (Abid et al., 2017). Therefore, DLIP has more potential applications than the other two methods, for applications such as icephobic metallic surfaces in aeronautics (Vercillo et al., 2020).

Targeted on metal substrates, a series of superhydrophobic surfaces have been achieved using DLIP or combined laser structuring methods. Pure aluminum (e.g., Al2024) substrates have also been processed to obtain either microgratings or micropillar arrays with a 7.0-μm-period by a two-beam DLIP system (Milles et al., 2019). The incident lasers were IR pulsed lasers with a wavelength of 1,064 nm, a pulse duration of 10 ps, and a repetition rate of 1 kHz. Titanium substrate (Ti64) was also explored and structured into micropillars with spatial wavelengths of both 2.7 and 5.4 μm (Vercillo et al., 2020). It should be noted that it is also likely that the LIPSS will be formed simultaneously during the DLIP process (Vercillo et al., 2020), as shown by Figures 1e–h. The LIPSS with nanoscale spatial wavelengths have enriched the complexity of DLIP-patterned hierarchical structures and hence give rise to the overall surface roughness and superhydrophobicity. Besides metals, other substrates can also be structured by DLIP for conveniently obtaining superhydrophobic surfaces, including polymers (Alamri and Lasagni, 2017), graphene oxide (Jiang et al., 2014, 2018; Ma et al., 2020), carbon fiber reinforced plastics (CFRP) (Hauschwitz et al., 2020), polycarbonate-sheets (Alamri et al., 2018), etc. Figures 1i–k show an typical example of DLIP-enabled patterning on 2D materials, i.e. bio-inspired graphene oxide surfaces with anisotropic wettability (Jiang et al., 2014). All of the substrates mentioned could be transformed into superhydrophobic surfaces using simple geometrical modifications, with the exception of CFRP.

## Direct Laser Writing (DLW)

Direct laser writing (DLW) is the most commonly used structuring high energy. DLW can define any 3D structures on a variety of material surfaces (e.g., femtosecond laser DLW) (Wu et al., 2014; Sun et al., 2015; Ma et al., 2017). Despite its outstanding 3D patterning ability, the technique has limitations due to low throughput, especially when dealing with large-area processing (Wu et al., 2014). As mentioned previously, the bioinspired superhydrophobic surfaces discussed here should ideally be capable of being applied to a large area. To achieve this, instead of adopting a single method, one could potentially combine DLW with other laser structuring methods to generate hierarchical surface micro-nanostructures. A coarse and fast scan of DLW could be utilized to define microgratings or microgrids, followed by nanoscale structuring via LIPSS or DLIP. For example, DLW and LIPSS were used together for a two-step fabrication on both a Ti-6Al-4V alloy (Huerta-Murillo et al., 2019) and stainless steel (Florian et al., 2018), which resulted in hierarchical surface structures composed of nanoripples and surrounding microgrids. The incident lasers for each step were 355-nm nanosecond lasers and 1,032-nm fs lasers [with similar examples have been discussed in section Direct Laser Interference Patterning (DLIP)].

DLW can also be utilized together with DLIP to form very complex 3D micro-nanostructures. This strategy would involve using DLW first, to define micropillar arrays. Then, DLIP could be performed to “carve” nanotrenches over the surface of the micropillars, as realized on an Al substrate in another study, which obtained extreme surface roughness as shown in Figure 1l (Milles et al., 2019). Nanosecond lasers were also adopted to structure aluminum alloy substrate together with a scanning step by a defocused laser beam (Hauschwitz et al., 2019). The two step processing finally realized hierarchical structures composed of micropillar arrays and nanoscale protrusions on top of them. DLW structuring for superhydrophobicity has been performed on polymers as well. As shown in Figures 1m,n, carbonization of polyimide films was realized by CO_2_ lasers to directly induced graphene arrays. Both the surface structure morphology and wettability were found dependent on the pulsing energy of CO_2_ lasers and high pulsing energy enabled superhydrophobicity (Nasser et al., 2020).

## Conclusion and Outlook

This minireview has introduced three laser patterning methods. These methods could be used to make geometrical modifications to various materials in realizing bioinspired superhydrophobic surfaces. Fast scan by DLW enables micro–structuring (such as microgratings or microgrids), which is convenient for constructing hierarchical surface structures, and can be easily combined with the other two methods discussed above. DLIP can directly acquire periodical 3D micro-nanostructures and simply manipulate surface wettability. Moreover, LIPSS can be self-organized during the structuring process of either DLW or DLIP with an even smaller scale (a spatial period of hundreds of nanometers). Since the three laser-structuring methods compensate for one another in either processing speed or structure scale, combined laser-surface-patterning is more advantageous in large-area-processing, particularly bioinspired superhydrophobic surfaces. Future studies might consider using more light sources (besides the infrared) for processing. The phase modulation of laser beams might also further increase the structuring speed. Comprehensive further exploitation of these laser-processing techniques will most likely continue to improve current surface modification methods, enabling bio-functionalization.

## Author Contributions

All authors have made a substantial and intellectual contribution to this minireview. ML contributed predominantly in this work.

## Conflict of Interest

The authors declare that the research was conducted in the absence of any commercial or financial relationships that could be construed as a potential conflict of interest.
